# Psychological distress among public and private healthcare professionals during the COVID-19 pandemic

**DOI:** 10.47626/1679-4435-2024-1227

**Published:** 2024-11-14

**Authors:** Giovanna Costa Guimarães, Gabrielle Silva Pereira, Helen Cristina Neves Trindade Rodrigues dos Santos, Elaine Cristine da Conceição Vianna, Thaís Alencar Linhares Peixoto, João Silvestre Silva-Junior, Cristiane Helena Gallasch

**Affiliations:** 1 School of Nursing, Universidade do Estado do Rio de Janeiro (UERJ), Rio de Janeiro, RJ, Brazil; 2 Graduate Program in Nursing, UERJ, Rio de Janeiro, RJ, Brazil; 3 Department of Medicine, Centro Universitário São Camilo, São Paulo, SP, Brazil; 4 School of Medicine, Universidade de São Paulo, São Paulo, SP, Brazil

**Keywords:** occupational health, occupational health surveillance, covid-19, mental health, mental disorders, saúde ocupacional, vigilância em saúde do trabalhador, covid-19, saúde mental, transtornos mentais

## Abstract

**Introduction:**

Healthcare workers in both the public and private systems were on the front line of the fight against the COVID-19 pandemic. As a consequence, they faced uncertainty, heavy demand, excessive working hours, and the fear of contracting the virus.

**Objectives:**

To investigate the occurrence of psychological distress among public and private healthcare workers at the start of the COVID-19 pandemic.

**Methods:**

An observational, quantitative study was conducted from April to June of 2020, enrolling workers who had cared for suspected and confirmed cases of covid-19 in Brazil. Psychological distress was assessed using the Self-Reporting Questionnaire-20. Associations between the outcome and independent variables were analyzed using the chi-square test.

**Results:**

Majorities of the 400 healthcare workers studied were nursing professionals (69.8%), worked in the Southeast region of Brazil (69.5%), were affiliated to a public healthcare institution (71.8%), and had a mean working week of 45.81 hours. The rate of psychological distress in the sample was 56.8%. There was no statistically significant association between the outcome and the type of organization (public/private). There were associations between psychological distress and professional category and between psychological distress and prior comorbidities.

**Conclusions:**

There was evidence of impact on mental wellbeing irrespective of care level or setting. Psychological distress was one of the greatest challenges faced by the healthcare workers during the pandemic, irrespective of the type of institution, and was a phenomenon of relevance to occupational health in general during the pandemic.

## Abstract (pt)

**Introdução:**

Os trabalhadores de saúde das redes pública e privada estiveram na linha de frente no combate à pandemia de covid-19, com consequente enfrentamento de incertezas, altas demandas, cargas horárias excessivas e medo de contrair o vírus.

**Objetivos:**

Investigar a ocorrência de sofrimento mental entre trabalhadores da assistência à saúde pública e privada no início da pandemia de covid-19.

**Métodos:**

Estudo observacional quantitativo, realizado entre abril e junho de 2020, incluindo trabalhadores que atuaram no atendimento aos casos suspeitos e confirmados de covid-19 no território brasileiro. O sofrimento mental foi avaliado por meio do Self-Reporting Questionnaire. Foi analisada a associação entre o desfecho e as variáveis independentes por meio do teste do qui-quadrado.

**Resultados:**

Entre os 400 profissionais de saúde, a maioria era da enfermagem (69,8%), atuantes na região Sudeste do país (69,5%), vinculados a uma instituição na rede pública de saúde (71,8%) e com jornada de trabalho média de 45,81 horas semanais. A taxa de sofrimento mental foi de 56,8% no grupo. Não houve associação estatisticamente significativa entre o desfecho e o tipo de organização (pública/privada). Houve associação entre sofrimento mental, categoria profissional e relato de comorbidades prévias.

**Conclusões:**

Evidenciou-se que, independentemente do nível ou local de assistência, houve impacto no bem-estar mental. O sofrimento mental foi um dos grandes desafios enfrentados pelos trabalhadores de saúde durante a pandemia, independentemente da natureza da instituição, sendo um fenômeno relevante à saúde do trabalhador em geral na situação de pandemia.

## INTRODUCTION

From the industrial revolution up to the intense process of social change that occurred in the Western World during the second half of the twentieth century, the relationships between health and work underwent great change, molding new aspects of relevance to occupational health, which now involves processes including multidisciplinary training, efforts to connect the different elements of the Unified Health System (SUS - Sistema Único de Saúde), and interinstitutional support, involving public universities in particular, and social control.^1,2^

Work, as an eminently social activity, plays a fundamental role in the living conditions of human beings, with positive effects when it meets workers’ basic needs, but also constituting a source of exposure to environmental risks, which are capable of directly impacting on their health.^3^

The hospital environment is one of the few environments in which all existing risks are found and can be defined as an unhealthy workplace. Chemical, biological, psychosocial, physical, and mechanical loads are all described in studies of healthcare settings, especially with regard to nursing workers, and are responsible for emotional attrition and for the occurrence of accidents and health problems.^4-6^

Hospital workers, especially those involved in direct provision of care, are exposed to a range of different loads and to the processes of attrition resulting from them, because of the need to move patients and heavy equipment, because of the physical attrition caused by the pace, organization, and division of work, because of living with pain and death, and because of contact with carriers of infectious diseases, all of which causes attrition of varying types.^7^ As a result, the relationships between work and health among healthcare workers have been the subject of countless studies, which have identified wide-ranging impacts on health, of both a negative and a positive nature.

In March of 2020, the global outbreak of the disease caused by the novel coronavirus (COVID-19) drove health services to a new level of activity, striving for safe provision of healthcare, focused on the many different professionals involved in caring for the population and dealing with a disease in which contagion is favored by close and unprotected contact with secretions or excretions from an infected patient, primarily via saliva droplets, and which causes patients to suffer severe respiratory insufficiency.^8-10^

Overcrowding of healthcare units and insufficient beds to admit the numbers of patients and a lack of the equipment needed to care for them aggravated the organization of work and impacted on the health of healthcare team members during the pandemic.^11-14^ In addition to these issues, failures to provide worker protection also stood out, a situation that was observed in many different countries.^10,12,13^ In this scenario, contamination and infection of the professionals involved in caring for patients was commonplace, with increasing numbers of cases of sickness and death among healthcare workers infected with COVID-19, already in 2020.^15^

Brazilian and international studies showed that the causes of the significant increases in risk of infection and sickness among healthcare workers were associated with scarcity or inadequate use of personal protective equipment (PPIs), inadequate or insufficient training in use of PPIs and in implementation of protective measures, prolonged exposure to large numbers of infected patients, intense pace of work, overload, absence of rest breaks, intense demand for treatment, and absence of mechanisms for supervision and monitoring of healthcare workers, in addition to psychological distress.^15^

Analyzing the causes mentioned above, it can be observed that, in addition to physical sickness, they also cause psychological distress and attrition through exposure of workers to longer shifts and with more intense work, without the necessary training and supervision.

Considering the dimensions of the pandemic within society, this is a subject that is relevant to planning and discussion of actions for the protection of the individual and collective health of people working at the different levels of health care (primary, secondary, tertiary, and quaternary), and at the different levels of care complexity, in addition to the characteristics of the institutions to which they are affiliated.

In Brazil, despite the significant efforts made by the SUS to deal with the crisis situation caused by the pandemic, defining policies and actions and providing direct care for suspected and confirmed cases, it proved a challenge to expand and improve the availability of beds, in the face of the historic underfunding to which the service had been subjected. The private system also faced these difficulties, although less effort was exerted to expand the number of beds, which also amplified the challenge for the SUS of dealing with the situation, considering the resources demanded and the need to support the workers involved.^16^

The objective of this study was to investigate the occurrence of psychological distress among public and private healthcare workers at the start of the COVID-19 pandemic.

## METHODS

A descriptive study with a quantitative approach was conducted according to the items on the STrengthening the Reporting of OBservational studies in Epidemiology (STROBE) checklist.

During the initial period of the COVID-19 pandemic, from April to June of 2020, health professionals in the whole of Brazil were invited to take part. Participants were identified using the snowball technique, which is a non-probabilistic sampling method, with limitations, but one that is appropriate for research involving groups that are difficult to access or study, and also when there is no precise information on the size of the group.^17^ Members of the research team provided suggestions for the first potential participants, who then suggested subsequent participants.

Professionals were eligible to answer the survey if they identified themselves as healthcare workers working on the frontline of the COVID-19 pandemic during the data collection period.

This study analyzed data from members of professions trained to provide bedside care, irrespective of the level of their qualifications, including nurses, nursing technicians, physicians, physiotherapists, psychologists, nutritionist, pharmacists, social workers, dentists, and speech and hearing therapists. Respondents were excluded if they were not in a healthcare profession or if they did not fill out all of the forms.

After reading and agreeing to the free and informed consent form, participants were asked to complete the sociodemographic, professional, and clinical data collections forms and the Self-Reporting Questionnaire (SRQ-20) online, using the Google^®^ forms platform. The survey was accessed using links sent by the research team members via e-mail or WhatsApp^®^.

The sociodemographic and clinical variables encompassed age and region of Brazil of residency/work, profession, number of institutional affiliations, weekly working hours, care level of job role, type of institutional affiliation, report of prior comorbidities and symptoms, and report of positive COVID-19 test. All of these data, with the exception of those directly related to provision of care for COVID-19, which could have happened at any time, were related to characteristics that must be true of the professionals and/or their jobs at the time of data collection.

The SRQ-20 instrument, used to screen for the dependent variable, was initially developed as a psychiatric screening instrument and has been validated for assessment of non-psychotic disorders, also known as common mental disorders (CMD). It comprises 20 questions and seven or more positive responses are indicative of a state of psychological distress, irrespective of the sex of the respondent.^18^

Data were tabulated in spreadsheets using Microsoft Excel^®^, version 16.0.12527.20986, and analyzed with the Statistical Package for Social Sciences (IBM SPSS^®^), version 22.

Numerical data were presented using descriptive statistics (frequencies, means, and standard deviations) and categorized for analysis. Categorical independent variables were analyzed using the chi-square test for association with the outcome psychological distress, considering that there was a statistical significant association if p was < 0.05.

The chi-square test was used to compare observed and expected frequencies of the categorical variables in the study, testing for independence of samples, i.e., associations between them, but without establishing relations of causality.^19,20^

The research protocol complies with National Health Council (Conselho Nacional de Saúde) Resolution 466/2012 and supplementary resolutions. It was registered on the Plataforma Brasil under Ethics Appraisal Submission Certificate 30599420.0.00000008 and the study was initiated after approval by the National Research Ethics Commission (CONEP - Comissão Nacional de Ética em Pesquisa), under protocol number 3.979.223, on April 18, 2020. All relevant ethical principles were respected, ensuring the legitimacy, privacy, and confidentiality of data and keeping participants anonymous, only publishing the results of the study.

## RESULTS

After the study was publicized, 436 workers completed the forms made available online, 400 of whom met the inclusion criteria for the study. The data collection strategy employing non-probabilistic sampling with the snowball technique had the effect that only those who wished to take part accessed the system, with no means of identifying refusals. There were no incomplete forms among the cases analyzed.

Data for the characteristics of the sample are shown in Table 1.

**Table 1:** Sociodemographic, occupational, and clinical characteristics of healthcare professionals caring for suspected/confirmed COVID-19 cases in the public and private healthcare systems (n = 400), Brazil, 2020

Variable	Mean (SD)	n	f (%)
Age (years)	38.03 (9.92)		
18-29		88	22.0
30-39		147	36.8
40-49		110	27.5
50-59		44	11.0
60 or over		11	2.8
Professional category			
Nursing		279	69.8
Medicine		65	16.3
Physiotherapy		22	5.5
Psychology		13	3.3
Nutrition		6	1.5
Social work		5	1.3
Pharmacy		5	1.3
Dentistry		4	1.0
Speech/hearing therapy		1	0.3
Region where working			
Southeast		278	69.5
Midwest		74	18.5
Northeast		26	6.5
South		17	4.3
North		5	1.3
Number of institutions at which employed			
One		240	60.0
Two		119	29.8
Three		23	5.8
Four		10	2.5
More than four		6	1.5
Working week (hours/week)	45.81 (15.88)		
Up to 39		98	24.5
40-60		250	62.5
More than 60		45	11.3
Type of institution			
Public		287	71.8
Private		66	16.5
Philanthropic		6	1.5
Non-governmental organization		2	0.5
More than one institution of different types		36	9.0
Care level			
Primary		125	31.2
Secondary		67	16.8
Tertiary		122	30.5
Quaternary		17	4.3
More than one level		63	15.8
Reported prior comorbidity			
No		169	42.3
Yes		140	35.0
Information not provided		92	22.8
Reported symptoms of COVID-19			
No		312	78.0
Yes		88	22.0

SD = standard deviation.

**Figure 1:**
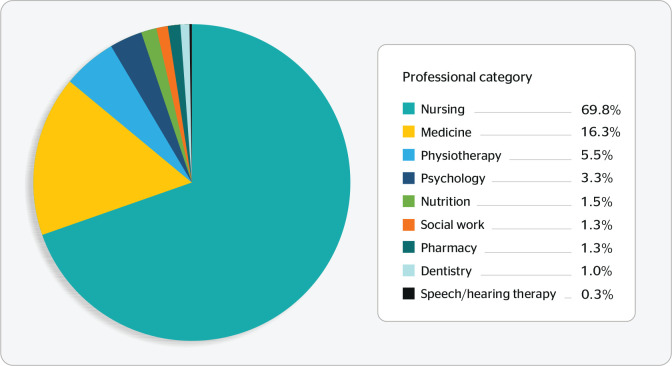
Distribution of professional categories reported by the participants, Brazil, 2020.

There was a predominance of young adults aged 30 to 39 years (36.8%) or 40 to 49 years (27.5%), of nurses (69.8%), and of workers from the Southeast region (69.5%). Although nursing predominated, the full distribution of respondents’ professional categories is illustrated in Figure 1.

Respondents who reported working for just one institution predominated, with a working week of 40 to 60 hours (62.5%), mean of 45.81 (±15.88) hours. It was observed that 71.8% reported working for the public healthcare system, while 16.5% stated they worked for private institutions. It was found that 35% of the interviewees reported a prior comorbidity, while just 22% mentioned having had symptoms of COVID-19.

Table 2 presents the 227 cases with psychological distress, which accounted for more than half of the sample (56.8%), distributed by categories of variables. There were statistically significant associations between psychological distress and age group, professional category, report of prior comorbidities, and report of symptoms of COVID-19. Of note were the large proportions of cases in the 30 to 39 years age group (66.7%) and the physiotherapist professional category (81.8%).

**Table 2:** Analysis of associations between psychological distress and independent variables, Brazil, 2020

Variable	Psychological distress				p^*^
	Yes		No		
	n (%)	%	n (%)	%	
Age (years) (n = 400)					0.004
18-29	49	55.7	39	44.3	
30-39	98	66.7	49	33.3	
40-49	58	52.7	52	47.3	
50-59	20	45.5	24	54.5	
60 and over	2	18.2	9	81.8	
Professional category (n = 400)					0.017
Nursing	165	59.1	114	40.9	
Medicine	29	44.6	36	55.4	
Physiotherapy	18	81.8	4	18.2	
Psychology	4	30.8	9	69.2	
Nutrition	4	66.7	2	33.3	
Social work	2	40.0	3	60.0	
Pharmacy	4	80.0	1	20.0	
Dentistry	1	25.0	3	75.0	
Speech/hearing therapy	0	0.0	1	100.0	
Region where working (n = 400)					0.866
Southeast	162	58.3	116	41.7	
Midwest	38	51.4	36	48.6	
Northeast	15	57.7	11	42.3	
South	9	52.9	8	47.1	
North	3	60.0	2	40.0	
Number of institutions at which employed (n = 398)					0.841
One	133	55.4	107	44.6	
Two	69	58.0	50	42.0	
Three	13	56.5	10	43.5	
Four	6	60.0	4	40.0	
More than four	5	83.3	1	16.7	
Working week (hours/week) (n = 393)					0.051
Up to 39	52	53.1	46	46.9	
40-60	137	54.8	113	45.2	
More than 60	33	73.3	12	26.7	
Type of institution (n = 397)					0.671
Public	160	55.7	127	44.3	
Private	38	57.6	28	42.4	
Philanthropic	3	50.0	3	50.0	
Non-governmental organization	2	100.0	0	0.0	
More than one institution of different types	23	63.9	13	36.1	
Care level (n = 394)					0.378
Primary	70	56.0	55	44.0	
Secondary	33	49.3	34	50.7	
Tertiary	74	60.7	48	39.3	
Quaternary	13	76.5	4	23.5	
More than one level	34	54.0	29	46.0	
Reported prior comorbidity (n = 309)					0.015
No	84	49.7	85	50.3	
Yes	89	63.6	51	36.4	
Reported symptoms of COVID-19 (n = 400)					0.003
No	165	52.9	147	47.1	
Yes	62	70.5	26	29.5	
Total	227	56.8	173	43.3	

* Chi-square test.

No dependence was observed between psychological distress and working region (p = 0.866), number of institutions (p = 0.841), working week (p = 0.051), type of institution at which respondent came into contact with cases (p = 0.671), or care level of job role (p = 0.378).

No statistically significant association was detected between psychological distress and working in the public or private sector.

## DISCUSSION

The objective of this study was to investigate the occurrence of psychological distress among public and private healthcare workers at the start of the COVID-19 pandemic. Psychological distress occurred in more than half of the sample, particularly among those who work at more than one institution, of different types. No statistically significant association was observed between psychological distress and type of institution.

The frequency of cases of psychological distress was higher than the rate observed in a study with professionals on the front-line of the COVID-19 pandemic working at a public service in the South of Brasil^21^ and higher than rates observed in previous studies in pandemic scenarios.^22-24^ There are a number of hypotheses that could explain this result, such as, for example, the period of data collection. The first 6 months of 2020 were the start of the pandemic, characterized by lack of in-depth information on the disease, which impacted workers’ physical and mental health, shortages of protective equipment, risk of contamination, sickness, and transmission of the COVID-19 virus, and changes to work environments, processes, and relationships in response to the pandemic.^25^ Another hypothesis for the high frequency of psychological distress is related to the method, which could have led to selection bias, with participation of professionals who were more interested in the subject.

The failure to observe statistically significant differences between those working in the public and private sectors indicates that these workers were similarly exposed to the same phenomenon. In a pandemic setting, everybody may have experienced psychological distress and it is possible that these symptoms persist.

It is of note that, as data were being collected, the numbers of public beds available were being increased and there was no information on the installed capacity in private hospitals.^16^ The predominance of participants working in the public sector may be linked to this context of expansion and, also, to greater availability and interest in participation among these workers, although it should be noted that there were more beds at SUS institutions during the study period.

The greater proportion of nursing team members may be related to the initial links sent out via the researchers’ network, but it should also be mentioned that this is the largest group of workers employed by health services.^26^ This participation also has a direct influence on the results for age of participants, since the profile of Brazilian nursing is a population of young adult workers, which is compatible with what was observed here.^27^

The considerable psychological distress observed in this category may have been identified because of the significant difference in the number of participants, but there are countless studies in the literature that report members of this profession having continuous and significant mental repercussions during this period, with potential for serious repercussions, including anxiety, depression, career abandonment, and even suicide.^28-31^

It was not possible to draw additional inferences with regard to the other healthcare professionals because of the heterogeneous nature of the sample, which is a limitation. Nevertheless, it is necessary to expand diagnosis and health surveillance of the other professionals, considering the peculiarities of the jobs of each member of the multidisciplinary team.

In the context of COVID-19, multidisciplinary work was essential for provision of initial care, continued care, and rehabilitation care to the people who had been infected. However, the professionals working in these scenarios, whether for one or more different employers, were constantly susceptible to physical and mental sickness, since they were working incessantly to meet the intense demand for care.^32^

The greater prevalence of participants from the Southeast region of Brazil among the study participants may have been related to the concentration of healthcare professionals in centers where healthcare technology is concentrated, or may even have been because it corresponds to the researchers’ areas of origin, where data collection started.

However, the literature also shows that there was a greater concentration of beds reserved for severe cases of COVID-19 in the Southeast region, which also illustrates the care inequalities that exist in Brazil, considering both human resources and installed infrastructure.^16,33^

It is considered of relevance to emphasize the participation of individuals with comorbidities in this study. During the period analyzed, it had already been observed that certain groups were at greater risk of developing complications if they were exposed to the novel coronavirus, but there was still resistance from employers to removing them from the front line of care provision^34^ and difficulties with reassignment because of the lack of human resources available.^35^

According to the literature, detection of associations between psychological distress and comorbidities reflects, in addition to overload, the influence of fear of sickness, of having to miss work, of overloading other team members, of failing to provide for the family, and even of dying.^25^

## CONCLUSIONS

It was possible to observe that the COVID-19 pandemic had a powerful impact on the mental health of professionals working on the front line of care provision, especially those in the nursing team, from the Southeast of Brazil, and with comorbidities. No significant differences were identified between the level of psychological distress among people working in the public sector and the private sector, but there were statistically significant associations between age group and professional category and detection of psychological distress.

There is a need to maintain surveillance of these data and for strategies developed by the workers and their workplaces to deal with this phenomenon.
